# Overview of human endogenous retroviruses found in human genome assembly GRCh37/hg19

**DOI:** 10.1186/1742-4690-10-S1-P6

**Published:** 2013-09-19

**Authors:** Luana Vargiu, Patricia Rodriguez-Tomé, Göran O Sperber, Enzo Tramontano, Jonas Blomberg

**Affiliations:** 1 Dept. of Life and Environmental Sciences, Cagliari, Sardinia, Italy; 2 Center for Advanced Studies, Research and Development, Pula, Sardinia, Italy; 3 Dpt of Neuroscience, Uppsala University, Uppsala, Sweden; 4 Medical Sciences, Uppsala University, Uppsala, Sweden

## Background

Human Endogenous Retroviruses (HERVs) are inherited after ancient germ-line cell infections by exogenous retroviruses. No replication-competent HERVs are known but few of them have one or several intact retroviral genes and retain some physiological effects. The number and classification of human endogenous retroviruses vary according to method of enumeration [1]. The focus of our project is to perform a systematic analysis and a classification of HERVs, in order to better understand their evolution and their involvement in shaping the human genome.

## Material and methods

Human genome (GRCh 37/hg19) was analyzed with RetroTector (ReTe) version 1.01 [2]. Rete was run on an Intel based machine, with 4 Xeon processors with 6 2.66 GHz cores, 256 Gb of RAM with an estimated execution time for the genome of 1 -2 days. The final classification of the 3,290 detected proviral chains was performed by BLASTing the concatenated Gag, Pro, Pol (as reconstructed by ReTe) aminoacid and proviral nucleotides against retroviral consensus and reference sequences and against RepeatMasker library of May 2013. Integration pattern analysis was made by custom algorithms.

## Results

The 3,290 proviral chains were classified in 57 unique groups which could be placed into class I (gamma-and epsilonlike), II (betalike) and III (spumalike). A few were more similar to errantiviruses. Integration patterns analysis showed a tendency for proviruses from the same clade to occur together, within 100 000 bases, maybe due to local duplications. Representatives from some gammaretroviral clades (HERVH and HERVE) integrated more frequently than expected by chance into the 5’ end of transcriptional units, mostly in antisense. Likewise, some gammaretroviral clades integrated more often in proximity of or within CpG islands. A few IncRNAs were found to contain HERV sequences. Thus, *cis*-effects from HERVs are to be expected. ReTe predicted open or nearly open reading frames for Gag, Pro, Pol and Env proteins for 30, 1024, 13 and 33 HERVs, respectively, but no provirus had all four in a completely open form. HML2, HERVH, HERVW, HERVFC and HERVT had at least one open of the four frames.

## Conclusions

We conclude that HERVs detected via a model-based search algorithm can be classified according to similarity with reference and consensus sequences. Recombinant forms seem to be more common than previously appreciated. However, phylogenetic trees displayed a consistent topology. HERV stop codon usage revealed mechanisms behind their gradual decay. Some gammaretroviral clades were more frequently integrated next to promoters and CpG islands than other HERV clades.

## References

[B1] Mayer J Blomberg J Seal RL A revised nomenclature for transcribed human endogenous retroviral loci Mobile DNA 2011 2 7 10.1186/1759-8753-2-7 21542922 PMC3113919

[B2] Sperber GO Airola T Jern P Blomberg J Automated recognition of retroviral sequences in genomic data--RetroTector Nucleic Acids Research 2007 35 4964 4976 10.1093/nar/gkm515 17636050 PMC1976444

